# Supplementary material for: Does Quality of Care (QoC) Perception Influence the Quality of Life (QoL) in Women with Endometriosis? Results from an Italian Nationwide Survey during Covid Pandemic
Source: Int J Environ Res Public Health. 2022 Dec 29;20(1):625. doi: 10.3390/ijerph20010625 (PMC9819574; doi:10.3390/ijerph20010625)
Supplement: Supplementary file 1 [file ijerph-20-00625-s001.zip › ijerph-2089698-supplementary.pdf]

Quality of care and perception of quality of life in women with endometriosis in the Covid era

This questionnaire is intended for women aged ≥18 years, diagnosed with endometriosis

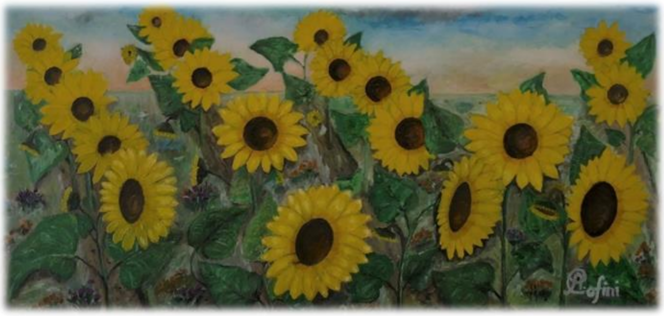

Dear Mrs,

*We propose that you participate in a survey to evaluate the impact of the Covid-19 Pandemic on the quality of care received and on the quality of life of women diagnosed with endometriosis, one year after the beginning of the Pandemic. To this end, we ask that you be available for about 10 minutes, inviting you to answer an anonymous online questionnaire. The data collected will be treated according to current legislation for the protection of privacy, will be processed in aggregate form not attributable to any participant and will be used for scientific work. We therefore invite you to give your consent after reading the information sheet on the study and the information note on data processing. You can interrupt your participation at any time, without needing to provide any explanation, thus obtaining the NOT use of the data.*____

Personal data

Q1 –years *

____

Q2- Nationality *

- Italian
- other __________

Q3 – Municipality of residence *

____

Q4 – Province *

Indicate the acronym with capital letters

____

Q5 - Region of origin *

Q6 - Marital status *

- married
- unmarried
- widow
- divorced
- cohabitant

Q7 - Do you live alone? *

- yes
- no

Q8 - If not, with how many other people (besides you)?

____

Q9 - Educational qualification *

- elementary school certificate

- middle school certificate
- Diploma
- Degree
- Postgraduate

Q10 - Indicate your height * Express the height in cm reporting only the number (example 165)

____

Q11 - Indicate your weight * Indicate the weight in kg reporting only the number (example 60)

____

Q12 - Do you currently have a job? *
o Yes, indefinitely
o Yes, for a fixed term
o No, I'm unemployed
o No, I'm a student
o No, I'm a housewife
o No, I'm retired

Q13 - Do you do physical activity? *
o Yes, moderate physical activity
o Yes, intense physical activity
o No, I am not currently physically active
o I have never exercised in my life

Q14 - Have you had one or more pregnancies? *
o Yes, in the past
o Yes, I'm currently waiting
o no
o other:___________________

Q15 - Smoking habit: currently you: *
o Smoke cigarettes (tobacco)
o Smokes an electronic cigarette
o No, but you have smoked in the past
o No, she has never smoked

Q16 - Do you ever drink a unit of alcohol (1 glass of beer or 1 glass of wine or spirits)? *
o Yes, only during meals (Skip to question Q16.1)
o Yes, mainly during meals (Skip to question Q16.1)
o Yes, mainly between meals (Skip to question Q16.1)
o Yes, only between meals (Skip to question Q16.1)
o I don't drink alcohol (Skip to question Q17)

Q16.1 - In a day, how many units of alcohol (1 glass of beer or 1 glass of wine or spirits) do you drink on average? *

____

Q16.1.1 - When you occasionally drink, how many alcoholic units (1 glass of beer or 1 glass of wine or spirits) do you consume on the same occasion? *

____

Q17 - What age did you find out you had endometriosis? *

____

Q18 - How many years after the onset of symptoms did you get the diagnosis? (if it took place after months, indicate zero years) *

- ___
- I have never had symptoms

Q19 - Have you ever been visited by centers/doctors specialized in endometriosis? *

- Yes (Skip to question Q19.1)
- No (Skip to question Q20)
- I don't remember/I don't know (Skip to question Q20).

Q19.1 - These centers/doctors are * (Select all applicable items)
- Public
- Private individuals with an agreement
- Private

Q19.2 - In the last 12 months, how many times have you been visited at these centers/doctors? * (Enter the number of times in "Other", reporting only the number (example: 2) (one answer only)
- Never (Skip to question Q20).
- other:______________________

Visits in the last 12 months to specialized centers/doctors

Q19.3 - During one of these visits, were you examined in depth, with ultrasound and gynecological examination? *
- yes
- no
- I don't remember/I don't know

Q19.4 - If yes, when was the last time it was visited in this way?

- In the last 6 months
- More than 6 months aSkip
- I don't remember

Q20 - In the last 12 months how many times have you seen your family doctor for reasons related to endometriosis? *
Enter the number of times in "Other", reporting only the number (example: 2) (one answer only).
- Never (Skip to question Q21).
- other:__________________

Q20.1 How satisfied are you with the care you received from your GP from 0 to 5?
(0=very dissatisfied, 1=dissatisfied, 2=fairly satisfied, 4=satisfied, 5=very satisfied) *
Only answer this question if you have visited your family doctor in the last 12 months. (one answer only).
- 1
- 2
- 3
- 4
- 5

Q21 - In the last 12 months, have you seen any specialists for problems related to endometriosis? *
o Yes (Skip to question Q21.1)
o No (Skip to question Q22)
o I don't remember (Skip to question Q22)

Q21.1 - Which specialist has seen you in the last 12 months for problems related to endometriosis? * (You can choose one or more answers. Select all applicable items)
□ Endocrinologist
□ Gastroenterologist
□ Proctologist
□ Urologist
□ Radiologist

□ Pain specialist (pain therapy)
□ Physiatrist
□ Physiotherapist
□ Sexologist
□ Psychologist
□ Nutritionist
□ Other:_________________

Q21.1.1 Add any other specialists who have seen you in the past 12 months for endometriosis-related issues
____

Q21.2 - Who sent you to the specialist? * (You can choose one or more answers. Select all applicable items)
□ Another specialist
□ Family doctor
□ You took the initiative

Q22 -In the last 12 months have you been hospitalized for problems related to endometriosis, excluding day hospital? *
o yes
o no
o I don't remember/I don't know

We would now like to ask you a few questions about tests and medicines that have been prescribed or recommended by your doctor or endometriosis center and questions about surgical interventions.

Q23 - Do you currently take drugs, prescribed or recommended by your gynecologist, for the treatment of endometriosis (such as estrogen-progestogen pills, progestogen pills, GNRH analogues)? *
- o yes
- o no

Q24 - Do you take other medicines not prescribed or recommended by a doctor, for problems related to endometriosis? * (you can select more than one answer)

□ Yes, painkillers
□ Yes, supplements
□ Yes, homeopathic products
□ No, I only take the medicines prescribed or recommended by my gynecologist
□ No, I am not taking any medications
□ Other:___________________

Q25 - Do you have one or more of the following complications due to endometriosis? *
(You can select one or more answers. Add in "other" further, possible, complications.)

□ I have no complications
□ Chronic pelvic pain
□ Dyspareunia (pain during intercourse)
□ Pelvic floor problems
□ Need for self-catheterization
□ Neuropathy/nerve problems
□ Infertility
□ Hysterectomy
□ Salpingectomy
□ Ovariectomy
□ Intestinal stricture
□ Intestinal resection
□ Intestinal stoma/urostomy
□ Bladder resection
□ Adherence syndrome
□ Other:

Q25.1 Add further complications, if any
____

Q26 - Have you ever had surgery for endometriosis and/or endometriosis-related complications? *

- □ Yes (Skip to question Q26.1)
- □ No (Skip to question Q27)

Q26.1 - What type of intervention? * (You can cross out multiple responses if you've had multiple interventions)

□ Diagnostic laparoscopy (to confirm or exclude pathology)
□ Operative laparoscopy (to carry out a treatment. Example removal of cysts, lysis of adhesions, etc.)
□ Laparotomy
□ I don't remember
□ Other:__________________________

We would like to ask you if you have received any information about your rights as an endometriosis patient

Q27 - Have you been informed about the benefits to which a patient suffering from endometriosis is entitled (for patients in the more advanced clinical stages, i.e. "moderate or III degree" and "severe or IV degree" the right to take advantage of an exemption for some specialist control services)? *
o Yes (Skip to question Q27.1)
o No (Skip to question Q28)
o I don't remember (Skip to question Q28).

Q27.1 - From whom did you receive the information? * (You can tick multiple answers)

□ From the family doctor
□ From the endometriosis centre/doctor
□ From associations of people with endometriosis
□ From family, friends
□ From other sources (district or health workers, pharmacists, journalists, radio, television, advertising, etc.)
□ I don't remember

The questions that follow refer to the structure that followed it more closely

Q28 - In the last 12 months, which structure followed your illness more closely? *

o Specific center or doctor specializing in endometriosis (Skip to question Q28.1)
o General practitioner's practice (Skip to question Q28.1)
o No structure (Skip to question Q28.1a)
o Other:_____________(Skip to question Q28.1)

Q28.1 - How do you think the structure's hours are? *

o excellent
o Adjust
o Just adequate
o Inadequate

Q28.2 - How accessible are the premises where it is visited, for example architectural barriers, lack of an elevator, steps, a lot of walking, etc.? *

o Very accessible
o Fairly accessible
o Accessibility with some difficulty
o not very accessible

Q28.3 - How do you judge the level of cleanliness and pleasantness? *

- o Optimum
- o Good
- o Sufficient
- o Not sufficient

Q28.4 How do you consider the anti-Covid preventive measures applied by the facility? *

- o Optimum
- o Good
- o Sufficient
- o Not sufficient

Q28.5 - During your last visits, how did you find the courtesy and availability of those who assisted you? *

- o Optimum
- o Good
- o Sufficient
- o Not sufficient

Q28.6 - During the last visits, how many times have things been explained to you in a way that you understand? *

- o Always
- o Often
- o sometimes
- o Never

Q28.7 - During your last visits to the clinic, how many times did you have the impression of being listened to attentively? *

- o Always
- o Often
- o sometimes
- o Never

Now I would like to ask you a few questions in particular about the LAST visit to the facility that followed you most closely

Q28.8 - How much time has passed from the booking to the visit? *
o Maximum one month
o Between one and six months
o Between six and 12 months
o Over a year

Q28.9 - What kind of visit was it? *
o New visit or control visit, rescheduled due to problems related to the Covid pandemic
o New visit
o Check-up visit

Q28.10 - The last time you visited; how long did it take to reach the facility? *
o Less than 15 minutes
o Between 15 minutes and 30 minutes
o Between 30 minutes and an hour
o More than an hour

Q28.11 - How long did you have to wait before they took care of you? *
o Less than 15 minutes

o Between 15 and 30 minutes
o Between 30 minutes and an hour
o More than an hour

Q28.12 - How long did your last check-up last from arrival to exit from the outpatient facility? *
Specify hours and/or minutes
____

Q28.13 - During the last visit, were you given an appointment for a specific date for the next visit? *

o yes
o No, I was told to call later to make an appointment
o No, I was told they will call me to fix the date
o No, I have not made an appointment
o I don't remember

Q28.14 - Overall, how do you rate the service that has been offered to you in the last 12 months? *
- o Optimum
- o Good
- o Sufficient
- o Not sufficient

Q28.15 - How do you rate the level of coordination between all the services and the different professionals dealing with your disease? *
- o Optimum
- o Good
- o Sufficient
- o Not sufficient

Q28.16 - What is the source of information that you consider most useful for following and understanding your disease? *

o The endometriosis center or my gynecologist
o The family doctor
o Patient associations
o I interpret all documents myself
o Websites
o social networks
o I don't have a truly effective source of information

Q28.17 - Do you think that in the last year in which we were affected by the Covid pandemic, compared to previous years, there have been changes in the management of your illness with reference to the following aspects? *

	Increased	*Decreased*	*Unchanged*
Waiting times from booking to visit			
Time taken to reach the facility			
Waiting times before the visit			

Q28.18 - Do you think that in the last year in which we were affected by the Covid pandemic, compared to previous years, there have been changes in the management of your disease with reference to the following aspects? *

	Improved(s)	Worsened(s)	Unchanged(s)
Facility opening hours			
Accessibility to the structure			
Cleanliness and pleasantness			
Courtesy and availability of those who assisted her			
Understandability of explanations			
Attention in listening			

Q28.19 - Do you think that the changes indicated can be attributed to Covid? *
o yes
o no

Q28.20 - If not, to what?
____
After Q28.20 question Q29.

If in the last 12 months, it has not been followed by any structure

Q28.1a - How many months have passed since the last visit? *
____

Q28.2a - Why haven't you been followed by a structure in the last 12 months? *

□ By personal choice linked to the fear of the Covid contagion
□ Because she was unable for health reasons related to Covid
□ You already have an appointment for a visit
□ She was followed in telemedicine
□ The visit has been cancelled/postponed due to issues related to the pandemic
□ Other:__________________________

Q29 - In relation to all the topics we have discussed, what would you propose to improve the assistance of women suffering from endometriosis in your Region?
____
____

Q30 - Do you suffer from other pathologies besides endometriosis? *

- yes
- no

Q31 - If you answered yes, you could indicate which ones if you wish
____

Q32 - Are you vaccinated for Covid-19? *

- yes
- no, not yet
- no, she does not intend to get vaccinated

Q33 - How did you find this survey? *

o Private group on Social Network
o Public group on Social Network
o Post on the wall of a Facebook contact
o Instagram
o Other Social Network
o Instant messaging services (WhatsApp, Telegram, Messanger, etc.)
o Association of women with endometriosis
o Email
o Other _________________

SF36

The questionnaire is concluded, you can click on "submit". We thank you for your cooperation

Qualità dell'assistenza e percezione della qualità della vita nelle donne con endometriosi in era Covid

Il presente questionario è destinato a Donne con età ≥18 anni, con diagnosi di endometriosi

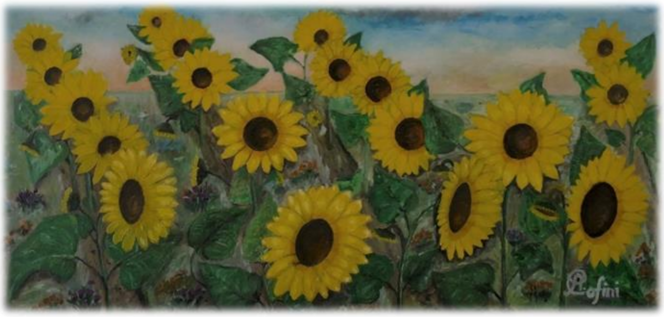

Gentile Signora,

Le proponiamo di partecipare ad un'indagine per valutare l'impatto della Pandemia Covid-19 sulla qualità dell'assistenza ricevuta e sulla qualità della vita delle donne con diagnosi di endometriosi, ad un anno dall'inizio della Pandemia. A tale scopo Le chiediamo la disponibilità di 10 minuti circa, invitandola a rispondere ad un questionario online anonimo. I dati raccolti saranno trattati secondo la normativa vigente per la tutela della privacy, saranno elaborati in forma aggregata non riconducibile ad alcun partecipante e saranno utilizzati per lavori scientifici. La invitiamo pertanto a dare il suo consenso dopo aver letto il foglio informativo sullo studio e la nota informativa sul trattamento dei dati. Potrà interrompere la sua partecipazione in qualsiasi momento, senza bisogno di fornire alcuna spiegazione ottenendo così il NON utilizzo dei dati

Dati anagrafici

D1 – Anni compiuti *

____

D2- Nazionalità *

- o Italiana
- o Altro __________

D3 – Comune di residenza *

____

D4 – Provincia *

Indicare la sigla con lettere maiuscole

____

D5 - Regione di provenienza *

- o Abruzzo
- o Basilicata
- o Calabria
- o Campania
- o Emilia-Romagna
- o Friuli Venezia Giulia
- o Lazio
- o Liguria
- o Lombardia
- o Marche
- o Molise
- o Piemonte
- o Puglia
- o Sardegna
- o Sicilia
- o Toscana
- o Trentino-Alto Adige

- Umbria
- Valle d'Aosta
- Veneto

D6 - Stato civile *
- Coniugata
- Nubile
- Vedova
- Divorziata
- Convivente

D7 - Vive da sola? *
- Si
- No

D8 - Se no, con quante altre persone (oltre Lei)?
____

D9 - Titolo di studio *
- Licenza elementare
- Licenza media
- Diploma
- Laurea
- Post laurea

D10 - Indichi la Sua altezza *
Esprimere l'altezza in cm riportando solo il numero (esempio 165)
____

D11 - Indichi il Suo peso *
Indicare il peso in kg riportando solo il numero (esempio 60)
____

D12 - Attualmente ha una occupazione? *
- Sì, a tempo indeterminato
- Sì, a tempo determinato
- No, sono disoccupata
- No, sono una studentessa
- No, sono casalinga
- No, sono pensionata

D13 - Svolge attività fisica? *
- Sì, attività fisica moderata
- Sì, attività fisica intensa
- No, non svolgo attività fisica attualmente
- Non ho mai svolto attività fisica nella mia vita

D14 - Ha avuto una o più gravidanze? *
- Sì, in passato
- Sì, sono attualmente in attesa
- No
- Altro:____________________

D15 - Abitudine al fumo: attualmente Lei: *
- Fuma sigarette (tabacco)
- Fuma sigaretta elettronica
- No, ma ha fumato in passato
- No, non ha mai fumato

D16 - Le capita di bere una unità alcolica (1 bicchiere di birra o 1 bicchiere di vino o un superalcolico)? *

- Sì, solo durante i pasti (Passa alla domanda D16.1)
- Sì, prevalentemente durante i pasti (Passa alla domanda D16.1)
- Sì, prevalentemente fuori dai pasti (Passa alla domanda D16.1)
- Sì, solo fuori dai pasti (Passa alla domanda D16.1)
- Non bevo alcolici (Passa alla domanda D17)

D16.1 - In un giorno quante unità alcoliche (1 bicchiere di birra o 1 bicchiere di vino o un superalcolico) mediamente beve? *

____

D16.1.1 Quando le capita di bere, quante unità alcoliche (1 bicchiere di birra o 1 bicchiere di vino o un superalcolico) consuma nella stessa occasione?

____

Qualità dell'assistenza ricevuta

D17 - A che età ha saputo di avere l'endometriosi? *

____

D18 - Dopo quanti anni dalla comparsa dei sintomi ha avuto la diagnosi? (se è avvenuta dopo mesi indichi zero anni) *

- 0
- 1
- 2
- 3
- 4
- 5
- 6
- 7
- 8
- 9
- 10
- più di 10
- Non ho mai avuto sintomi

D19 - È mai stata visitata da centri/medici specializzati in endometriosi? *

- Si (Passa alla domanda D19.1)
- No (Passa alla domanda D20)
- Non ricordo/non so (Passa alla domanda D20).

<u>Centri/medici specializzati in endometriosi</u>

D19.1 - Questi centri/medici sono *

Seleziona tutte le voci applicabili.

- Pubblici
- Privati convenzionati
- Privati

D19.2 - Negli ultimi 12 mesi quante volte è stata visitata presso questi centri/medici? (Indichi un numero) *

Inserire in "Altro" il numero di volte, riportando solo il numero (esempio: 2) *(una sola risposta)*

- Mai (Passa alla domanda D20).
- Altro:______________________

<u>Visite negli ultimi 12 mesi in centri/medici specializzati</u>

D19.3 - In una di queste visite, è stata visitata in maniera approfondita, con ecografia e visita ginecologica? *

- ○ Sì
- ○ No
- ○ Non ricordo/non so

D19.4 - Se sì, quando è stata l'ultima volta che è stata visitata in tale maniera?

- ○ Negli ultimi 6 mesi
- ○ Più di 6 mesi fa
- ○ Non ricordo

D20 - Negli ultimi 12 mesi quante volte ha visto il Suo medico di famiglia per motivi legati all'endometriosi? *
Inserire in "Altro" il numero di volte, riportando solo il numero (esempio: 2) *(una sola risposta).*

- ○ Mai (Passa alla domanda D21).
- ○ Altro:__________________

D20.1 Quanto si ritiene soddisfatta dell'assistenza ricevuta dal Suo medico di famiglia da 0 a 5?
(0=molto insoddisfatta, 1=insoddisfatta, 2=discretamente soddisfatta, 4=soddisfatta, 5=molto soddisfatta) *
Risponda a questa domanda soltanto se negli ultimi 12 mesi si è recata dal Suo medico di famiglia. *(una sola risposta).*

- ○ 1
- ○ 2
- ○ 3
- ○ 4
- ○ 5

D21 - Negli ultimi 12 mesi è stata visitata da qualche specialista per problematiche correlate all'endometriosi? *

- ○ Sì (Passa alla domanda D21.1)
- ○ No (Passa alla domanda D22)
- ○ Non ricordo (Passa alla domanda D22)

D21.1 - Quale specialista l'ha visitata negli ultimi 12 mesi per problematiche correlate con l'endometriosi? *
Può scegliere una o più risposte

Seleziona tutte le voci applicabili.

- ☐ Endocrinologo
- ☐ Gastroenterologo
- ☐ Proctologo
- ☐ Urologo
- ☐ Radiologo
- ☐ Algologo (terapia del dolore)
- ☐ Fisiatra
- ☐ Fisioterapista
- ☐ Sessuologo
- ☐ Psicologo
- ☐ Nutrizionista
- ☐ Altro:_________________

D21.1.1 Aggiunga eventuali altri specialisti che l'hanno visitata negli ultimi 12 mesi per problematiche correlate all'endometriosi
____

D21.2 - Chi l'ha inviata dallo specialista? *
Può scegliere una o più risposte

Seleziona tutte le voci applicabili.

- ☐ Altro specialista

☐ Medico di famiglia
☐ Ha preso Lei l'iniziativa

D22 - Negli ultimi 12 mesi è stata ricoverata in ospedale per problematiche legate all'endometriosi, escluso il day hospital? *

o Sì
o No
o Non ricordo

<u>Vorremmo farLe ora qualche domanda sugli esami e farmaci che Le sono stati prescritti o consigliati dal Suo medico o dal centro per l'endometriosi e domande sugli interventi chirurgici.</u>

D23 - Attualmente, assume farmaci, prescritti o consigliati dal Suo ginecologo, per la terapia dell'endometriosi (quali pillola estroprogestinica, pillola progestinica, analoghi del GNRH)? *

o Sì
o No

D24 - Assume altri farmaci non prescritti o consigliati da un medico, per problematiche correlate all'endometriosi? *
Può selezionare più di una risposta

☐ Sì, antidolorifici
☐ Sì, integratori
☐ Sì, prodotti omeopatici
☐ No, assumo soltanto i farmaci prescritti o consigliati dal mio ginecologo
☐ No, non assumo alcun farmaco
☐ Altro:___________________

D25 - Presenta una o più delle seguenti complicanze, dovute all'endometriosi? *
Può selezionare una o più risposte. Aggiungere in "altro" ulteriori, eventuali, complicanze.

☐ Non presento complicanze
☐ Dolore pelvico cronico
☐ Dispareunia (dolore durante i rapporti)
☐ Problemi al pavimento pelvico
☐ Necessità di autocateterismi
☐ Neuropatia/problemi ai nervi
☐ Infertilità
☐ Isterectomia
☐ Salpingectomia
☐ Ovariectomia
☐ Stenosi intestinale
☐ Resezione intestinale
☐ Stomia intestinale/urostomia
☐ Resezione vescicale
☐ Sindrome aderenziale
☐ Altro:

D25.1 Aggiungere ulteriori, eventuali, complicanze
____

D26 - Ha mai subito un intervento chirurgico per endometriosi e/o per complicanze correlate all'endometriosi? *

☐ Sì (Passa alla domanda D26.1)
☐ No (Passa alla domanda D27)

D26.1 - Che tipo di intervento? *

Può barrare più risposte se ha subito più interventi.

- ☐ Laparoscopia diagnostica (per confermare o escludere la patologia)
- ☐ Laparoscopia operativa (per effettuare un trattamento. Esempio rimozione di cisti, lisi di aderenze, ecc)
- ☐ Laparotomia
- ☐ Non ricordo
- ☐ Altro:___________________________

<u>Vorremmo chiederLe se ha ricevuto informazioni sui Suoi diritti come malata di endometriosi</u>
D27 - È stata informata sulle agevolazioni cui ha diritto una paziente affetta da endometriosi (per le pazienti negli stadi clinici più avanzati cioè “moderato o III grado” e “grave o IV grado” è riconosciuto il diritto ad usufruire di un’esenzione per alcune prestazioni specialistiche di controllo)? *
--

- o Sì (Passa alla domanda D27.1)
- o No (Passa alla domanda D28)
- o Non ricordo (Passa alla domanda D28).

D27.1 - Da chi ha ricevuto l'informazione? *
Può barrare più risposte

- ☐ Dal medico di famiglia
- ☐ Dal centro/medico specializzato in endometriosi
- ☐ Da associazioni di persone con endometriosi
- ☐ Da familiari, amici
- ☐ Da altre fonti (addetti del distretto o ASL, farmacisti, giornalisti, radio, televisione, pubblicità, ecc)
- ☐ Non ricordo

<u>Le domande che seguono si riferiscono alla struttura che l’ha seguita più da vicino.</u>
D28 - Negli ultimi 12 mesi, quale struttura ha seguito più da vicino la Sua malattia? *

- o Centro specifico o medico specializzato in endometriosi (Passa alla domanda D28.1)
- o Ambulatorio del medico di famiglia (Passa alla domanda D28.1)
- o Nessuna struttura (Passa alla domanda D28.1a)
- o Altro:_____________(Passa alla domanda D28.1)

<u>Struttura che ha seguito più da vicino la Sua malattia</u>
D28.1 - Come ritiene che siano gli orari della struttura? *

- o Ottimi
- o Adeguati
- o Appena adeguati
- o Non adeguati

D28.2 - Quanto sono accessibili i locali dove viene visitata, per esempio barriere architettoniche, mancanza di un ascensore, scalini, molto da camminare, ecc.? *

- o Molto accessibili
- o Abbastanza accessibili
- o Accessibilità con una certa difficoltà
- o Poco accessibili

D28.3 - Come giudica il livello di pulizia e gradevolezza? *

- o Ottimo
- o Buono
- o Sufficiente
- o Non sufficiente

D28.4 Come reputa le misure preventive anti-Covid applicate dalla struttura? *

- Ottime
- Buone
- Sufficienti
- Non sufficienti

D28.5 - Durante le Sue ultime visite, come Le sono sembrate la cortesia e la disponibilità di chi l'ha assistita? *

- Ottime
- Buone
- Soddisfacenti
- Non sufficienti

D28.6 - Durante le ultime visite quante volte Le sono state spiegate le cose in maniera a Lei comprensibile? *

- Sempre
- Spesso
- Qualche volta
- Mai

D28.7 - Durante le Sue ultime visite in ambulatorio, quante volte ha avuto l'impressione di essere ascoltata con attenzione? *

- Sempre
- Spesso
- Qualche volta
- Mai

<u>Vorrei farLe ora qualche domanda in particolare sull'ULTIMA visita nella struttura che l'ha seguita più da vicino</u>
D28.8 - Quanto tempo è trascorso dalla prenotazione alla visita? *

- Massimo un mese
- Tra uno e sei mesi
- Tra sei e 12 mesi
- Oltre un anno

D28.9 - Che tipo di visita era? *

- Nuova visita o visita di controllo, riprogrammata per problemi legati alla pandemia Covid
- Nuova visita
- Visita di controllo

D28.10 - L'ultima volta che è stata visitata, quanto tempo ha impiegato per raggiungere la struttura? *

- Meno di 15 minuti
- Tra 15 minuti e 30 minuti
- Tra 30 minuti e un'ora
- Più di un'ora

D28.11 - Quanto tempo ha dovuto attendere prima che si siano occupati di Lei? *

- Meno di 15 minuti
- Tra 15 e 30 minuti
- Tra 30 minuti e un'ora
- Più di un'ora

D28.12 - Quanto tempo è durato complessivamente il Suo ultimo controllo dall'arrivo fino all'uscita dalla struttura ambulatoriale? *
Specificare ore e/o minuti
____

D28.13 - Durante l'ultima visita, Le è stato dato un appuntamento ad una data precisa per la visita successiva? *

- Sì
- No, mi è stato detto di chiamare in seguito per fissare un appuntamento
- No, mi è stato detto che mi telefoneranno per fissarmi la data
- No, non mi è stato fissato un appuntamento
- Non ricordo

D28.14 - Globalmente come giudica il servizio che Le è stato offerto negli ultimi 12 mesi? *

-
- Ottimo
- Buono
- Sufficiente
- Non sufficiente

D28.15 - Come giudica il livello di coordinamento tra tutti i servizi e i diversi professionisti che si occupano della Sua malattia? *

- Ottimo
- Buono
- Sufficiente
- Non sufficiente

D28.16 - Qual è la fonte di informazioni che considera più utile per seguire e capire la Sua malattia? *

- Il centro per l'endometriosi o il mio ginecologo
- Il medico di famiglia
- Le associazioni di malati
- Interpreto da me stessa tutti i documenti
- Siti-web
- Social networks
- Non dispongo di una fonte di informazione veramente efficace

D28.17 - Ritiene che nell'ultimo anno in cui siamo stati colpiti dalla pandemia Covid, rispetto agli anni precedenti, ci siano stati cambiamenti nella gestione della sua malattia con riferimento ai seguenti aspetti? *

	**Aumentati**	**Diminuiti**	**Invariati**
Tempi di attesa dalla prenotazione alla visita			
Tempi impiegati per raggiungere la struttura			
Tempi di attesa prima della visita			

D28.18 - Ritiene che nell'ultimo anno, in cui siamo stati colpiti dalla pandemia Covid, rispetto agli anni precedenti, ci siano stati cambiamenti nella gestione della Sua malattia con riferimento ai seguenti aspetti? *

	**Migliorata/i**	**Peggiorata/i**	**Invariata/i**
Orari della struttura			
Accessibilità alla struttura			
Pulizia e gradevolezza			
Cortesia e disponibilità di chi l'ha assistita			
Comprensibilità delle spiegazioni			
Attenzione nell'ascolto			

D28.19 - Ritiene che i cambiamenti indicati possano essere attribuititi alla Covid? *

- ○ Sì
- ○ No

D28.20 - Se no, a cosa?
____

Se negli ultimi 12 mesi non è stata seguita da alcuna struttura

D28.1a - Quanti mesi sono trascorsi dall'ultima visita? *
____

D28.2a - Perchè negli ultimi 12 mesi non si è fatta seguire da una struttura? *

- ☐ Per scelta personale legata alla paura del contagio Covid
- ☐ Perché impossibilitata per motivi di salute legati alla Covid
- ☐ Ha già un appuntamento per una visita
- ☐ È stata seguita in telemedicina
- ☐ La visita è stata annullata/spostata per problematiche legate alla pandemia
- ☐ Altro:___________________________

D29 - In rapporto a tutti gli argomenti di cui abbiamo discusso, che cosa proporrebbe per migliorare l'assistenza delle donne malate di endometriosi della Sua Regione?
____
____

D30 - Soffre di altre patologie, oltre all'endometriosi? *

- ○ Sì
- ○ No

D31 - Se ha risposto sì, se vuole può indicare quali
____

D32 - È vaccinata per la Covid-19? *

- ○ Sì
- ○ No, non ancora
- ○ No, non intende vaccinarsi

D33 - Come ha trovato questo questionario? *

- ○ Gruppo privato su Social Network
- ○ Gruppo pubblico su Social Network
- ○ Post sulla bacheca di un contatto Facebook
- ○ Instagram
- ○ Altro Social Network
- ○ Servizi di messagistica istantanea (WhatsApp, Telegram, Messanger, ecc)
- ○ Associazione donne con endometriosi
- ○ Email
- ○ Altro __________________

STATO DI SALUTE SF36

Il questionario è concluso può cliccare su "invio". La ringraziamo per la collaborazione
